# Inflammation and Oxidative Stress Are Elevated in the Brain, Blood, and Adrenal Glands during the Progression of Post-Traumatic Stress Disorder in a Predator Exposure Animal Model

**DOI:** 10.1371/journal.pone.0076146

**Published:** 2013-10-09

**Authors:** C. Brad Wilson, Leslie D. McLaughlin, Anand Nair, Philip J. Ebenezer, Rahul Dange, Joseph Francis

**Affiliations:** 1 Department of Comparative Biomedical Sciences, School of Veterinary Medicine, Louisiana State University, Baton Rouge, Louisiana, United States of America; 2 Department of Pathobiological Sciences, School of Veterinary Medicine, Louisiana State University, Baton Rouge, Louisiana, United States of America; Universidade de São Paulo, Brazil

## Abstract

This study sought to analyze specific pathophysiological mechanisms involved in the progression of post-traumatic stress disorder (PTSD) by utilizing an animal model. To examine PTSD pathophysiology, we measured damaging reactive oxygen species and inflammatory cytokines to determine if oxidative stress and inflammation in the brain, adrenal glands, and systemic circulation were upregulated in response to constant stress. Pre-clinical PTSD was induced in naïve, male Sprague-Dawley rats via a predator exposure/psychosocial stress regimen. PTSD group rats were secured in Plexiglas cylinders and placed in a cage with a cat for one hour on days 1 and 11 of a 31-day stress regimen. In addition, PTSD group rats were subjected to psychosocial stress whereby their cage cohort was changed daily. This model has been shown to cause heightened anxiety, exaggerated startle response, impaired cognition, and increased cardiovascular reactivity, all of which are common symptoms seen in humans with PTSD. At the conclusion of the predator exposure/psychosocial stress regimen, the rats were euthanized and their brains were dissected to remove the hippocampus, amygdala, and pre-frontal cortex (PFC), the three areas commonly associated with PTSD development. The adrenal glands and whole blood were also collected to assess systemic oxidative stress. Analysis of the whole blood, adrenal glands, and brain regions revealed oxidative stress increased during PTSD progression. In addition, examination of pro-inflammatory cytokine (PIC) mRNA and protein demonstrated neurological inflammatory molecules were significantly upregulated in the PTSD group vs. controls. These results indicate oxidative stress and inflammation in the brain, adrenal glands, and systemic circulation may play a critical role in the development and further exacerbation of PTSD. Thus, PTSD may not be solely a neurological pathology but may progress as a systemic condition involving multiple organ systems.

## Introduction

Post-Traumatic Stress Disorder (PTSD) is an anxiety disorder that can develop in response to real or perceived life-threatening situations. According to the Diagnostic and Statistical Manual of Mental Disorders IV-Text Revision (DSM-IV-TR), a diagnosis of PTSD necessitates exposure to a life-threatening event, intrusive recollections of the event, avoidance of associated stimuli and numbing of general responsiveness, hyperarousal not present before the trauma, and a significant social impairment. All of these symptoms must also persist for at least 30 days[1]. To date, no definitive diagnostic biomarkers have been identified for PTSD. Recent research, however, points toward physiological abnormalities in the brain, hypothalamic-pituitary-adrenal (HPA) axis, and immune system that may be responsible for the psychological manifestations of the disorder[2]–[4]. Many chronic conditions such as hypertension, heart failure, and metabolic syndrome perpetuate in a state of increased inflammation and oxidative stress, exacerbating their pathophysiology[5]–[7]. We hypothesize that similar physiological mechanisms may play a role in PTSD development.

Exposure to psychologically traumatic events, such as those experienced during combat or other situations posing a legitimate threat to safety and survival, place individuals at significant risk for developing PTSD. A growing body of evidence suggests that exposure to traumatic stressors and subsequent psychological trauma may result in increased morbidity and premature demise of patients. Much of the data available suggest traumatic exposure and subsequent PTSD may lead to increased incidence of cardiovascular disease, diabetes, chronic fatigue syndrome, and other conditions[8]–[11]. Most of these diseases have detrimental inflammatory components that may exacerbate their progression. Inflammation is a critical component of the immune response, but acute and chronic inflammation can damage cellular mechanisms. Stressful events affect the immune system by reducing the cellular response to mitogen stimulation, decreasing production of natural killer cell activity and altering levels of cytokines. Cytotoxic T lymphocytes, which regulate the balance between Th1 and Th2 cells, are altered by stress leading to a Th2 dominant response, resulting in an unrestrained production of pro-inflammatory cytokines (PICs). These PICs, especially the interleukins, have been shown to play an important role in modulating disease processes. An important and detrimental consequence of increased cytokine production is the induction of nitric oxide (NO) and reactive oxygen species (ROS)[12], [13].

Elevated levels of PICs and ROS can cause cell death and tissue damage, although the cellular mechanisms responsible for initiating these processes during the stress response have remained poorly understood. In addition to leukocytic responses, PIC upregulation may also be due to the activation of inflammasomes[14]. Inflammasomes are multiprotein complexes that cooperate with pattern-recognition receptors (PRRs) such as Toll-like receptors (TLRs) and NOD (nucleotide oligomerization domain)-like receptors (NLRs). When the inflammasome complex is activated, it cleaves pro-caspase-1 into its active form, which results in, among other things, the production of PICs and initiates the inflammatory response. When proliferation of PICs exceeds the ability of local cellular receptors to utilize them in autocrine or paracrine functions, they become blood-borne. These cytokines can then be transported across the blood-brain barrier[15], where they activate microglial cells and induce the production of more cytokines. The process results in a positive feedback loop, which can become self-sustaining and cause systemic organ dysfunction. Research from our lab has demonstrated the damaging effects of PICs when quantities reach uncontrolled levels. We have also shown that blocking certain downstream transcription factors and gene modifiers of these cytokines reduces oxidative stress, inflammation, and associated damage in hypertension, heart failure (HF), and metabolic syndrome (MetS)[6], [7], [16]. In light of this information, this study investigates whether oxidative stress and inflammation increase in the brain, adrenal glands, and systemic circulation during the progression of PTSD using a predator exposure/psychosocial stress animal model.

## Materials and Methods

### Ethics Statement

This study was carried out in strict accordance with the recommendations of the Institute for Laboratory Animal Research's 2011 *Guide for the Care and Use of Laboratory Animals*, under the auspices of an animal care and use protocol approved by the Louisiana State University Institutional Animal Care and Use Committee (Protocol Number: 12-067).

### Animals

Naïve adult male Sprague-Dawley rats (Harlan Laboratories, Indianapolis, IN) were used in all experiments. The rats were the same age (12 weeks) and approximately the same weight (±15 g) upon delivery. Rats were pair-housed in standard plastic microisolator cages and had access to food and water *ad libitum*. The cages were maintained in ventilated racks (racks hold eight cages vertically and five horizontally) and each cage was randomly assigned to a specific rack location to ensure groups were evenly distributed. The vivarium room was kept on a 12-hour light/dark cycle (0700-1900), room temperature was maintained at 20±1°C, and humidity ranged from 23–42%. After a one-week acclimation period, the mean weight of all rats was 347.9 g±4.5. Two cats, one male and one female (Harlan Laboratories, Indianapolis, IN (male), and Tulane University, New Orleans, LA (female)) were used for all predator exposures. Cats were seven and ten years old, respectively. They were housed in an open room (15′×15′) in the vivarium with access to food, water, and enrichment devices *ad libitum*. The cat room was on the same light/dark cycle and maintained at similar temperature and humidity as the rat room.

### Stress Induction

**Figure 1:**
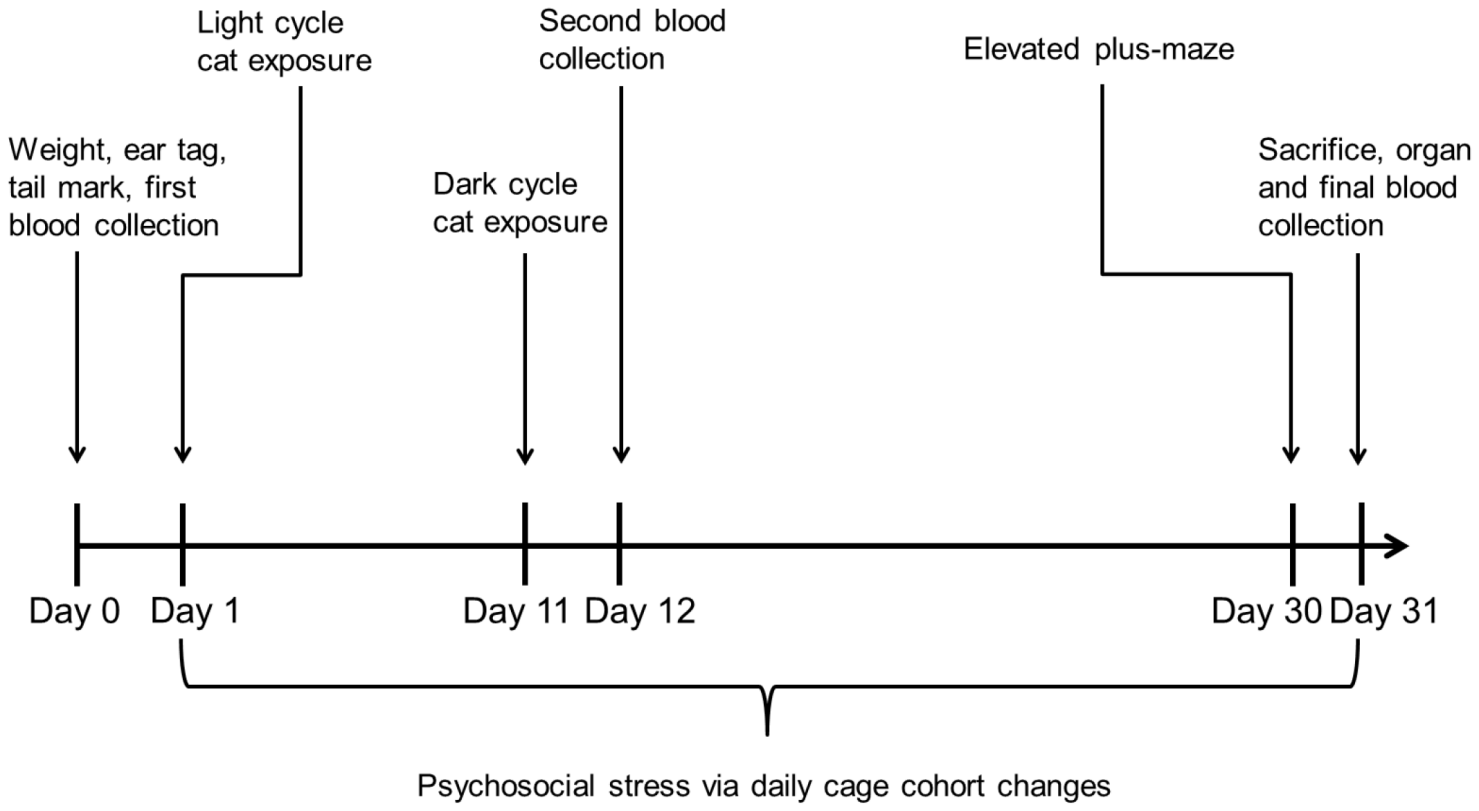
Predator exposure/psychosocial stress regimen. The predator exposure/psychosocial stress model includes two cat exposures over a 31-day period, combined with daily cage cohort changes. In addition, blood was collected at three different time points and ROS levels were measured to determine oxidative stress within groups and between groups. Anxiety was measured at the end of the stress regimen via EPM.

Following the acclimation period, rats were brought to the laboratory and under isoflurane anesthesia were weighed, ear-tagged, tail-marked (ear tag number written on tail for easy identification), and 250–500 µL of blood was drawn from either the tail or lateral saphenous vein. The rats were then randomly assigned to the “PTSD” or “control” group and returned to the vivarium for 24 hours. The following day, PTSD rats were started on a predator exposure/psychosocial stress regimen, published and validated by Zoladz, et al., designed to produce a pre-clinical PTSD that closely mimics signs and symptoms seen in human patients[17](Figure 1). Briefly, PTSD rats were individually isolated in cylindrical, Plexiglas containers (IITC Life Science, Inc., Woodland Hills, CA; tail cuff restraint containers for 400–600 g rats and Kent Scientific, Torrington, CT; tail cuff restraint containers for 300–500 g rats) and canned cat food (Friskies, Purina, St. Louis, MO) was smeared on the outside of the cylinders. The cylinders prevented direct contact with the cats, and the cat food induced predatory movement in the cats. Studies show a moving cat invokes a greater fear response than a sedentary cat[18]. Rats were then placed in a stainless steel holding cage (76 cm×76 cm×60 cm) consisting of a solid metal floor with a hinged, metal rod door, with a cat for one hour. The first cat exposure was conducted during the light cycle (0700-1900). Ten days later, a second cat exposure was conducted during the dark cycle (1900-0700). In addition to the cat exposures, starting on day one the rats were subjected to psychosocial stress by changing their cage cohort daily. The cage cohort rotation was established prior to the start of the experiment, whereby each rat was never housed with the same rat on consecutive days and never housed with the same rat more than four times in a month. The predator exposure/psychosocial stress regimen was continued for 31 days. After 31 days, PTSD and control group rats were euthanized via CO_2_ inhalation, perfused with a phosphate buffered vascular rinse solution, and the brains were removed. The hippocampus, amygdala, and pre-frontal cortex (PFC) were dissected and flash-frozen in liquid nitrogen. The thymus and adrenals glands were also removed, trimmed, weighed, and flash-frozen.

### Blood Collection

Approximately 250–500 µL blood per animal was drawn from either the lateral saphenous veins of rats in both groups, allowing a minimum of 24 hours between any consecutive blood draws to prevent anemia. Anesthesia was induced in an isoflurane anesthesia chamber and maintained on low-flow isoflurane via a nose cone throughout the procedure. The hind legs were shaved to allow access to the lateral saphenous veins. Blood was collected using a heparinized 22 g needle and microcentrifuge tube with 50 µL of heparin. Petroleum jelly (Vaseline, Unilever, Englewood Cliffs, NJ) was applied at the puncture site when necessary to reduce clotting. Alternatively, a lateral tail vein was used to collect blood after warming the tail in water and using the same gauge heparinized needle and microcentrifuge tube with 50 µL of heparin.

### Elevated Plus-Maze

Rats were placed in the center of the elevated plus-maze (EPM) (EB-Instruments (Bioseb), Tampa Bay, FL) facing an open arm and allowed to roam freely for five minutes. Movement was monitored via an overhead camera and captured with a specifically designed software program (BioEPM3C, EB-Instruments, Tampa Bay, FL). The primary measurements were the number of entries into each arm (total ambulations) and the total time spent in the open vs. closed arms. An arm entry was defined as all four feet crossing into a different arm. The stand for the EPM was approximately 36” above the floor, and each arm measured 11 cm×50 cm.

### Growth Rates and Organ Weights

At the beginning of the experiment and after the 31-day stress induction period, rats were weighed to determine average growth rate/day of PTSD and control animals. Following euthanasia, the adrenal glands and thymus were removed and weighed. Those organs were chosen, as their structure and function have been shown to be adversely affected by stress[19], [20]. The adrenal weights were combined as one weight for each animal, and weight was expressed as mg/100 g body weight. Thymus weight was also expressed as mg/100 g body weight.

### Corticosterone Analysis

Plasma corticosterone was measured using the DetectX Corticosterone ELISA (K-014-H1, Arbor Assays, Ann Arbor, MI). The samples were diluted and prepared as per the protocol, optical density was analyzed at 450 nm with a plate reader (VersaMax, Molecular Devices, Hayward, CA), and a standard curve was created based on the concentrations in each sample.

### Electron Paramagnetic Resonance Spectroscopy

Total ROS, superoxide, and peroxynitrite were measured in whole blood (baseline, day 12, and day 31), brain tissue (hippocampus and PFC), and the adrenal glands via electron paramagnetic resonance (EPR) as previously described[21], [22]. Blood was drawn from all rats at the beginning of the experiment (baseline), one day after the second cat exposure (day 12), and at the end of the predator exposure/psychosocial stress regimen (day 31). Superoxide, peroxynitrite, and total ROS levels in the blood were compared as repeated measures within the control and PTSD groups, and also between groups (control vs. PTSD), to analyze oxidative stress during PTSD progression. Analysis of oxidative stress between groups was also compared with tissue collected following euthanasia. Two different spin probes were used for EPR studies. 1-Hydroxy-3-methoxycarbonyl-2,2,5,5-tetramethyl-pyrrolidine(CMH) was used to measure tissue ROS and superoxide O2•−, and 1-hydroxy-3-carboxy-2,2,5,5-tetramethylpyrrolidine(CPH) was used for measurement of tissue peroxynitrite (OONO−). Briefly, pieces of tissue were incubated at 37°C with CMH (200 µM) for 30 min for ROS measurement; PEG-SOD (50 U/ µl) for 30 min, then CMH (200 µM) for an additional 30 min for O2•− measurement; or CPH (500 µM) for 30 min for OONO− measurement. Aliquots of incubated probe media were then taken in 50- µl disposable glass capillary tubes (Noxygen Science Transfer and Diagnostics) for determination of ROS, O2•−, or OONO− production. All EPR measurements were performed using an EMX ESR eScan BenchTop spectrometer and super-high quality factor microwave cavity (Bruker Company, Germany).

### Real-Time PCR Analysis

Semi-quantitative real-time RT-PCR (n = 6/group) was used to determine the mRNA levels of IL-1β and the NALP3 inflammasome in the hippocampus. The primer sequences used for real-time PCR are given (Table 1). In brief, the rats were euthanized using CO_2_ inhalation, perfused with a phosphate buffered solution directly into the left ventricle, and the brains were quickly removed, dissected, and immediately flash-frozen in liquid nitrogen. Total RNA isolation, cDNA synthesis and RT-PCR were performed as previously described[23]. Gene expression was measured by the ΔΔCT method and was normalized to GAPDH mRNA levels. The data is presented as fold change of the gene of interest relative to that of control animals.

**Table 1:** Rat primers used for real-time RT-PCR.

Gene	Sense	Antisense
GAPDH	agacagccgcatcttcttgt	Cttgccgtgggtagagtcat
IL-1β	cagaccactttggcagacttcact	Ggattcgttggctgttcggtcg
NALP3	cagaaggcatgtgagaagca	Tgggtgtagcgtctgttgag

IL, (Interleukin); NALP3, (NACHT, LRR, PYD domains containing protein 3); GAPDH, (Glyceraldehyde 3-phosphate dehydrogenase).

### Western Blot Analysis

Tissue homogenates from the hippocampus and PFC were subjected to Western Blot (WB) analysis (n = 10/group) for the determination of protein levels of IL-1β, the NALP3 inflammasome, and GAPDH. The extraction of protein and WB was performed as previously described[23]. The specific antibodies used included: IL-1β, NALP3, and GAPDH. Primary antibodies were commercially obtained: IL-1β and GAPDH, 1∶1000 dilution (SC-7884 and SC-20358 respectively, Santa Cruz Biotechnology, Santa Cruz, CA); and NALP3, 1∶1000 dilution (Biorbyt, San Francisco, CA.). Secondary antibodies were commercially obtained: anti-mouse, 1∶500 dilution and anti-rabbit, 1∶500 dilution (SC-2314 and SC-2004 respectively, Santa Cruz Biotechnology, Santa Cruz, CA). Immunoreactive bands were visualized using enhanced chemiluminescence (ECL Plus, Amersham), band intensities were quantified using ImageJ imaging software (NIH), and were normalized with GAPDH.

### Statistical Analysis

All data are presented as mean ± SEM. Statistical analysis was done by one-way ANOVA with a Tukey's post hoc test for multiple comparisons, and unpaired Student's T-tests were used for two-column analyses. P-values less than 0.05 were considered statistically significant. Statistical analyses were performed using Prism (GraphPad Software, Inc; version 5.0).

## Results

### Growth Rates and Organ Weights

The PTSD group displayed a significantly diminished growth rate over the 31-day stress period, t(18) = 2.78, p<0.05. The same group also showed an increase in adrenal gland weight, t(18) = 5.66, p<0.0001, and a decrease in thymus weight, t(18) = 4.81, p<0.0001 relative to the control group (Table 2).

**Table 2:** Growth rate and organ weights.

Group	Growth Rate (g/day)	Adrenal Wt. (mg/100 g.b.w.)	Thymus Wt. (mg/100 g.b.w.)
Control (n = 10)	1.72 (0.14)	10.73 (0.49)	80.24 (2.11)
PTSD (n = 10)	1.11* (0.17)	14.03* (0.32)	61.51* (3.28)

* p<0.05 relative to the control group.

### Plasma Corticosterone

**Figure 2:**
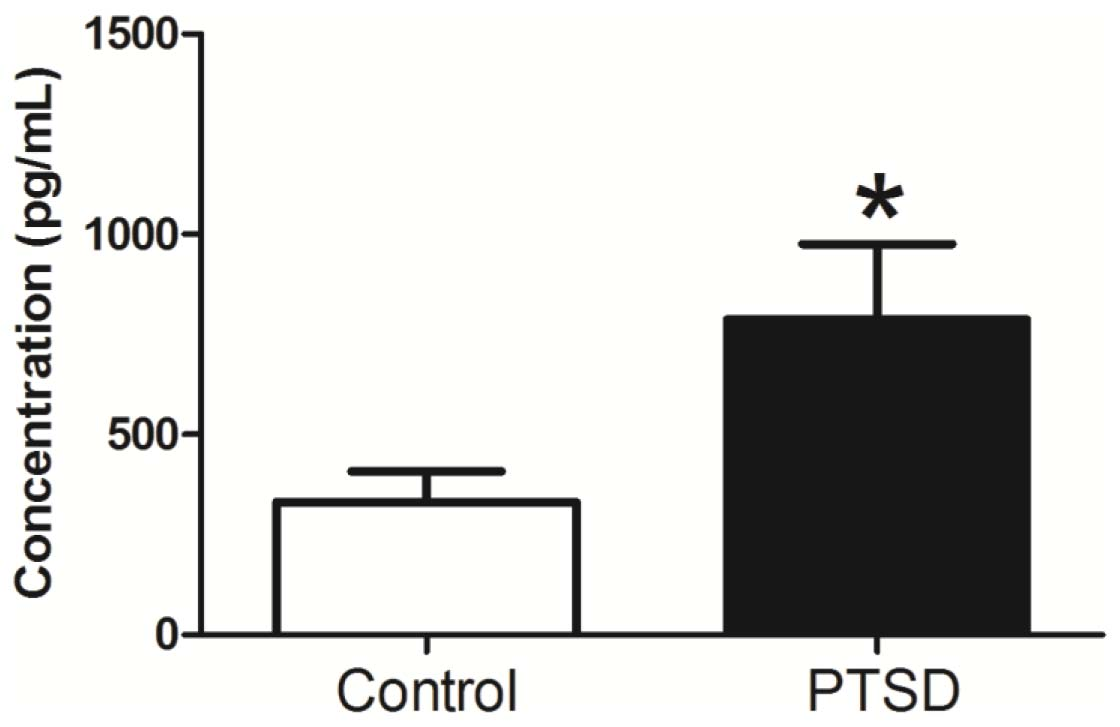
Post-stress corticosterone levels. After 31 days of the predator exposure/psychosocial stress regimen, plasma corticosterone levels were higher in the PTSD group. Corticosterone was measured in plasma collected at the time of sacrifice and frozen prior to testing. Data are presented as ± SEM. *p<0.05 relative to the control group.

The PTSD group displayed higher plasma corticosterone levels, t(14) = 2.24, p<0.05 relative to the control group (Figure 2).

### Elevated Plus-Maze Performance

**Figure 3:**
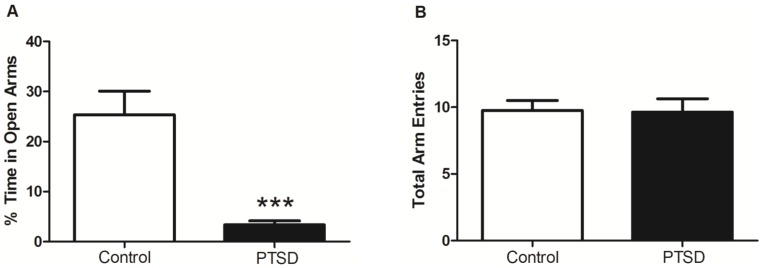
Elevated plus-maze performance. The PTSD group displayed significantly higher anxiety than the control group, as evidenced by their reluctance to spend time in the open arms of the EPM (A). Total ambulations, however, were essentially equal between the two groups (B). Anxiety on the EPM was tested within 24 hours of the final day of the 31-day stress regimen. Data are presented as mean ± SEM. ***p<0.0001 relative to the control group.

The comparison of anxiety levels revealed the PTSD group spent considerably less time in the open vs. closed arms, t(18) = 3.88, p<0.05. Overall ambulations, however, were not affected as both groups were still relatively active inside the maze, t(18) = 0.34, p>0.05 (Figure 3).

### Oxidative Stress Analysis

**Figure 4:**
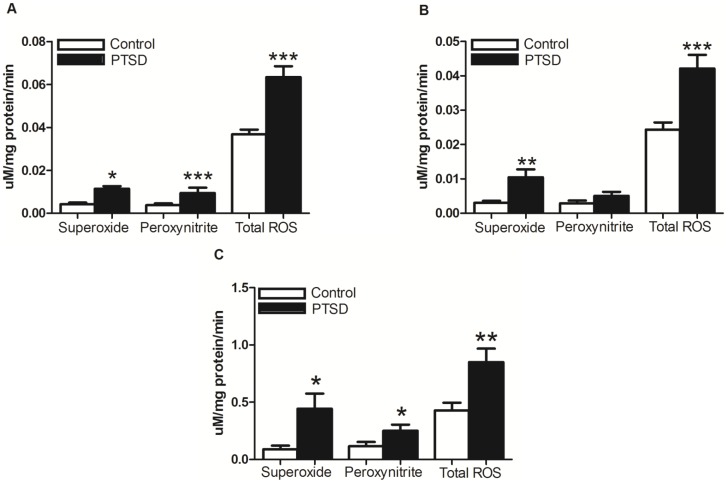
Reactive oxygen species in the brain and adrenal glands. Superoxide, peroxynitrite, and total ROS were all significantly elevated in the hippocampus (A) and adrenal glands (C) in the PTSD group. Superoxide and total ROS were also elevated in the pre-frontal cortex (B) in the PTSD group, but peroxynitrite did not reach significance. All data are presented as mean ± SEM. *p<0.05, **p<0.001, ***p<0.0001 relative to the control group.

**Figure 5:**
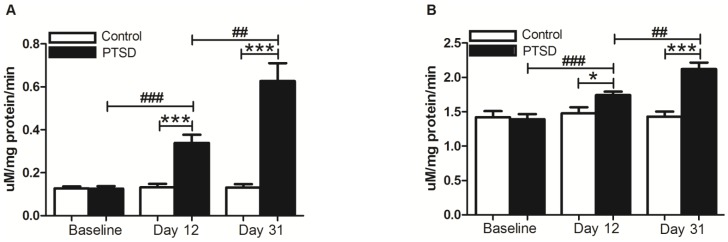
Reactive oxygen species in the blood. Superoxide (A) and total ROS (B) in the blood were measured between groups and within groups at three time points during the predator exposure/psychosocial stress regimen. Superoxide and total ROS were at approximately the same level for the PTSD and control groups at the beginning of the experiment, but progressively rose during stress. All data are presented as mean ± SEM. *p<0.05, ***p<0.0001 relative to the control group. ##p<0.001, ###p<0.0001 relative to the previous measurement of the same group.

To investigate the influence of the predator exposure/psychosocial stress regimen on oxidative stress/redox balance, we examined levels of superoxide, peroxynitrite, and total ROS in the hippocampus, PFC, and adrenal glands. In the hippocampus, PFC, and adrenal glands, analysis of the EPR data revealed superoxide, peroxynitrite, and total ROS were elevated in all three regions (p<0.05). Peroxynitrite in the PFC, however, did not reach significance (p>0.05) (Figure 4). In whole blood drawn at three different time points, superoxide levels were nearly identical at baseline, elevated at 12 days, and further elevated at 31 days relative to the control group (p<0.05) (Figure 5). Within-group comparison via repeated measures with the same animals of the PTSD group demonstrated superoxide levels were elevated (day 12 vs. baseline, p<0.05) and further elevated (day 31 vs. day 12, p<0.05). Repeated measures of total ROS levels yielded similar results (Figure 5). Repeated measures in the control group revealed superoxide and total ROS levels remained relatively unchanged (p>0.05).

### Brain Inflammatory Markers

**Figure 6:**
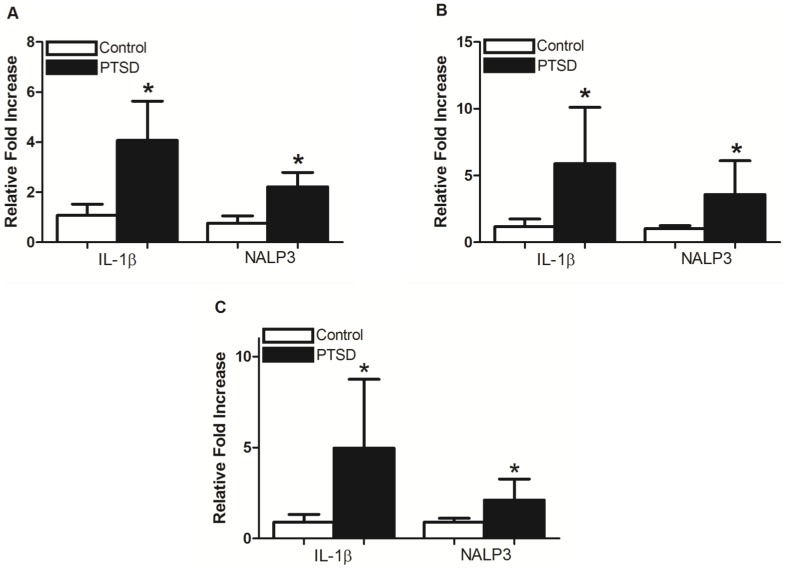
RT-PCR on the brain tissue. RT-PCR revealed IL-1β and NALP3 mRNA were significantly elevated in the hippocampus (A), PFC (B), and amygdala (C) in the PTSD group. All data presented as mean ± SEM. *p<0.05 relative to the control group.

**Figure 7:**
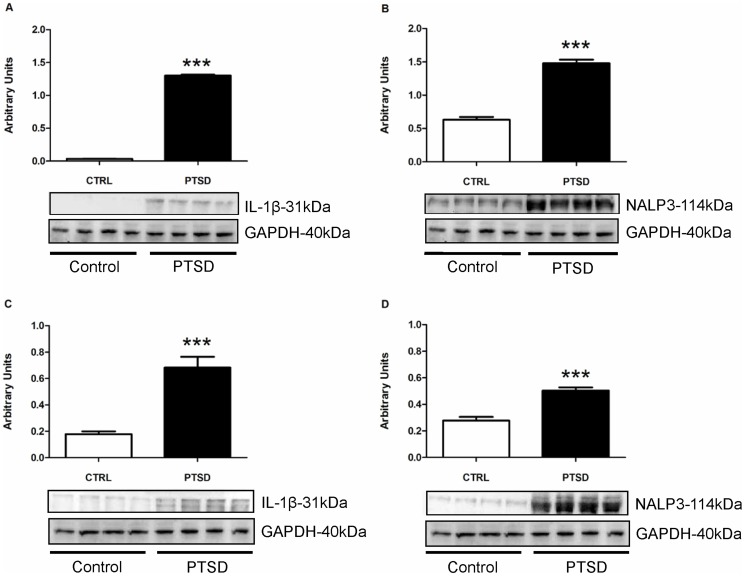
Western blot on the hippocampus (A &B) and PFC (C & D) tissue. Western Blot showed IL-1β and NALP3 protein in the hippocampus (A & B respectively) and PFC (C & D respectively) were significantly elevated in the PTSD group. All data presented as mean ± SEM. *p<0.05 relative to the control group.

To investigate the influence of the predator exposure/psychosocial stress regimen on inflammation, we examined mRNA (Figure 6) and protein (Figure 7) levels of IL-1β and NALP3 in the hippocampus, PFC, and amygdala. The PTSD group demonstrated significantly elevated mRNA levels of IL-1β and NALP3 in all three regions. In the hippocampus and PFC, protein for IL-1β and NALP3 were significantly higher in the PTSD group relative to controls.

## Discussion

The present study sought to analyze specific pathophysiological mechanisms involved in the progression of PTSD by employing a predator exposure/psychosocial stress regimen. Few animal models of PTSD exist, but the model by Zoladz et al. has been shown to cause heightened anxiety, exaggerated startle response, impaired cognition, and increased cardiovascular reactivity [17], all of which are common symptoms reported in humans with PTSD[24], [25]. Although animal models have their limitations, a major component missing from human PTSD research is the ability to ascertain physiological data prior to PTSD development and while the disorder is progressing. We have successfully obtained such data with this model, and to our knowledge we are the first to report the time-dependent progression of oxidative stress in the blood in PTSD animals. In addition, we discovered damaging ROS in the hippocampus, PFC, and adrenal glands were also upregulated in response to the predator exposure/psychosocial stress regimen. In the brain, mRNA and protein for cytokines and cytokine-producing mechanisms were significantly elevated, demonstrating a neuroinflammatory component in PTSD. Three novel and important findings emerged from this study. First, oxidative stress and inflammation are upregulated in the brain, namely the hippocampus, PFC, and amygdala, in response to psychological stress. Second, oxidative stress increases not only in the brain but also in the blood and adrenal glands, indicating PTSD may progress as a systemic condition involving multiple organ systems. Last, and possibly most important, oxidative stress increases in the blood in a time-dependent manner within the same group of animals.

Most of the current PTSD research is focused on human patients, which has its advantages and disadvantages. The advantages are self-evident, but disadvantages include the variable backgrounds among patients, the type of stressful event (e.g., combat, rape, kidnapping, etc.), reduced experimental control in treatment studies, and the inability to determine baseline physiological data before PTSD developed. An animal model mitigates those variables, but as there are multiple animal models used in anxiety disorder research with varied approaches and methods, careful selection was necessary. For our experiments, it was important to select an animal model of PTSD that matched, as closely as possible, the behavioral, psychological, and physiological elements of PTSD in humans. The predator exposure/psychosocial stress model by Zoladz et al., possesses both predictive and construct validity, meaning the model is sensitive to clinically effective pharmacologic agents and the rationale underlying the model displays similarities to human PTSD[26]. The model demonstrates three hallmark features of PTSD: hormonal abnormalities, a long-lasting traumatic memory, and persistent anxiety[17].

**Figure 8:**
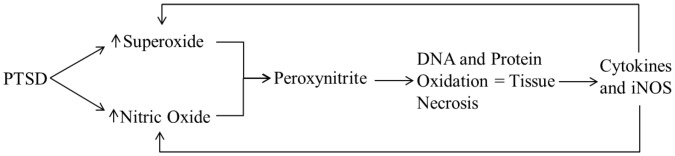
Reactive oxygen species cause tissue damage and necrosis. Cytokine production in PTSD leads to upregulation of superoxide and nitric oxide, which bind to form the very potent oxidizer peroxynitrite. The resulting tissue damage sustains the positive feedback loop causing further detrimental effects.

The roles of oxidative stress and inflammation in other pathological conditions including cardiovascular disease, diabetes mellitus, metabolic syndrome, and neurological diseases are well established[7], [23], [27], [28]. Reactive oxygen species, or free radicals, have unpaired valence shell electrons and cause damage by oxidizing proteins, lipids, nucleic acids, and other cellular components. They are produced naturally via mitochondrial leakage, xanthine oxidase, and other pathways, and they are important in cell signaling, homeostasis, and host defense. Under normal conditions, the body's antioxidant mechanisms (e.g., superoxide dismutase, glutathione peroxidase, uric acid) scavenge ROS and convert them to inert compounds. Oxidative stress, by contrast, occurs when there is an imbalance between naturally occurring ROS and the body's ability to convert them via antioxidants. As ROS levels increase, they can cause DNA and protein oxidation, leading to tissue necrosis and upregulation of PICs[27]. An important function of cytokines is to transmit information concerning inflammatory responses to the CNS[29]–[32]. The CNS then participates in negative feedback regulation of the peripheral immune response by releasing pituitary hormones (adrenocorticotropic hormone [ACTH] and arginine vasopressin [AVP]) and increasing sympathetic drive. Cortisol, AVP, and sympathetic nerve activity all act to suppress further peripheral production of cytokines, and cortisol also acts centrally to inhibit further production of corticotropin-releasing hormone (CRH). When cytokine upregulation exceeds cellular usage, however, they may then be transported across the blood-brain barrier[15], activate microglial cells, and induce the production of more cytokines. The process becomes a positive feedback loop which can become self-sustaining and result in severe organ dysfunction. A proposed pathway by which this pathophysiology may occur is presented in figure 8.

Recent research has sought to determine the pathophysiology of PTSD development and to identify diagnostic biomarkers, but progress has been slow on both endeavors. Glucocorticoid derangements have been found to be variable[3], [33], [34], hippocampus structural changes may or may not occur[2], [35], and studies have not been able to consistently delineate the specific roles of neurotransmitters[36] in the disorder. These conflicting reports indicate a need to explore other mechanisms possibly involved in PTSD pathophysiology. As mentioned previously, oxidative stress and inflammation are implicated in many disease processes. The involvement of oxidative stress and inflammation in PTSD, however, has only recently garnered attention. Oosthuizen et al., reported that PTSD was exacerbated by increased levels of NO and other ROS, causing cellular damage in the hippocampus[2]. Diehl et al., discovered DNA breakage, a sign of oxidative stress, in the hippocampus of rats subjected to stress via a maternal separation model[37]. Our results demonstrate ROS and PICs are significantly upregulated during the progression of PTSD, indicating an increase in oxidative stress and inflammation in the brain, adrenals, and systemic circulation. Whether or not the structural and functional damage these mechanisms may cause is contributory or a by-product of PTSD progression is yet to be determined.

Previous research regarding plasma glucocorticoid (cortisol in humans, corticosterone in rodents) levels in PTSD has been met with varied results. Numerous studies have shown PTSD patients to have lower cortisol levels than the general population[33], but there have also been studies reporting no difference[34] or elevated levels[3]. Cortisol is the primary hormone involved in the stress response, and its primary roles include gluconeogenesis and suppression of the immune system. Cortisol functions effectively in a narrow therapeutic range, and hypercortisolism (Cushing's syndrome) or hypocortisolism (Addison's disease) both have deleterious effects. In this experiment we found plasma corticosterone was elevated in the PTSD group vs. controls, which contrasted with the results of Zoladz et al., in this model[17]. In their experiment, lower baseline corticosterone levels were obtained in the undisturbed (non-dexamethasone/vehicle injected) group, whereas we measured corticosterone levels from plasma collected during euthanasia procedures immediately after the predator exposure/psychosocial stress regimen. The difference in results underscores the rapid nature of glucocorticoid changes in response to even the slightest stressor, and may provide a rationale for often inconsistent results reported in animal models of PTSD. Despite these differences, glucocorticoid abnormalities seem to play a legitimate role in PTSD progression, but to what extent still remains unanswered.

Chronic stress affects normal growth patterns, possibly due to increased HPA axis stimulation or other mechanisms acting in concert with upregulated glucocorticoids[38]. The decrease noted in body weight growth rates in our rats may be attributable to endocrine abnormalities similar to those seen in human PTSD patients. During traumatic or stressful events, there is a profound release of cortisol from the adrenal cortex[39]. The hypothalamic-pituitary-adrenal (HPA) axis is activated by the hypothalamus via corticotropin releasing hormone (CRH) at the median eminence, which stimulates the release of ACTH from the anterior pituitary gland. In turn, ACTH causes the release of cortisol from the adrenal cortex. In non-PTSD individuals, cortisol exerts negative feedback control at the hypothalamus and pituitary, but recent research indicates PTSD patients may display aberrant endocrine profiles[40], [41]. One such abnormality is enhanced negative feedback inhibition of the HPA axis, resulting in increased levels of CRH[42]–[44]. Higher levels of CRH can inhibit feeding behavior, even in food-deprived animals[38], [45]. Increased adrenal gland weight may also be due to excessive glucocorticoid production without proper negative feedback from the hypothalamus, resulting in adrenal hypertrophy and hyperplasia[20]. The substantive decrease seen in thymus weight may be a result of increased oxidative stress or cortisol toxicity causing thymocyte apoptosis[46].

One of the diagnostic criteria for PTSD is hyperarousal, which includes an exaggerated startle response and heightened anxiety[1]. To measure anxiety levels, we used the elevated plus-maze (EPM). Rodents have a natural tendency to explore novel environments, but open areas or alleys (without protective walls) invoke a greater fear and avoidance response[47]. The EPM is widely used as a measure to test fear or anxiety and has been extensively validated for use in rats[48], [49]. The EPM is a four-arm maze, shaped like a “plus” sign, that consists of two open arms and two closed arms. The premise behind the EPM is based on rodents' natural aversion of open spaces versus their desire to explore novel environments. Entry into the open areas is associated with increased freezing behavior as well as increased plasma corticosterone levels, indicating heightened anxiety[49]. Anxiogenic compounds or procedures can increase avoidance of the fear-provoking open arms, whereas anxiolytic compounds or procedures can increase open arm exploration[49]. The primary criteria correlated with anxiety levels are total arm entries (overall ambulations) and the percent time spent in the open vs. closed arms. We found that the predator exposure/psychosocial stress regimen had a significant anxiogenic effect regarding time spent in the open vs. closed arms. These findings demonstrate the model induced a marked increase in anxiety in the PTSD group vs. controls. The lack of difference in overall ambulatory activity between the groups suggests activity level and anxiety may be independent measures in animal models of PTSD.

In summary, this study sought to determine if, as in many other disease processes, oxidative stress and inflammation increased during PTSD progression. To answer this question, we used a predator exposure/psychosocial stress animal model of PTSD. We validated stress induction via an EPM and also found plasma corticosterone levels to be elevated in the PTSD group. In addition, growth rate and thymus weights were lower in the PTSD group, while adrenal gland weight was higher vs. controls. Our findings indicate ROS and PICs are upregulated in the adrenal glands, circulating blood, and the areas of the brain associated with PTSD, indicating an increase in oxidative stress and inflammation as the disorder progresses.

## Conclusion

We utilized a predator exposure/psychosocial stress animal model of PTSD to demonstrate how oxidative stress and inflammation may play a key role in PTSD development. We found ROS and PICs were elevated in all three regions of the brain commonly associated with PTSD, indicating increased oxidative stress and inflammation. In addition, oxidative stress and inflammation were elevated systemically, as evidenced by increased ROS and PICs in the adrenal glands and circulating blood. We also noted a time-dependent nature of oxidative stress by analyzing whole blood drawn at different time points. Levels of superoxide, peroxynitrite, and total ROS in the blood rose in an exponential nature throughout the stress period, while corresponding ROS levels in the control animals remained relatively constant. Our use of the model established by Zoladz et al.,[17] produced similar behavioral and physiological results, including a diminished growth rate, larger adrenal glands, a smaller thymus, and decreased time spent in the open arms of the EPM, confirming their results. In contrast to their findings, we obtained higher post-stress corticosterone levels. Their results, however, were obtained following a dexamethasone suppression test, whereas we tested plasma corticosterone on frozen blood collected immediately following euthanasia. Overall, our results demonstrate the progressive nature of oxidative stress and inflammation during PTSD development and may provide new targets for pharmacologic and non-pharmacologic treatments. Future studies by our lab will seek to elucidate neurotransmitter modulation in response to the predator exposure/psychosocial stress regimen and the subsequent response to various treatment modalities.
